# Occupational health, risk factors, and protection among unmanned aerial vehicle operator in the high-altitude region of China: an observational study

**DOI:** 10.3389/fpubh.2026.1764053

**Published:** 2026-04-01

**Authors:** Peilin Shu, Yuanjing Zheng, Xiaoxue Yan, Pengfei Wang, Wencong Li, Hongbo Jia, Minghao Yang

**Affiliations:** 1 Air Force Medical Center, Air Force Medical University, Beijing, China; 2 School of Biological Science and Medical Engineering, Beihang University, Beijing, China

**Keywords:** China, high-altitude region, occupational health, operator, unmanned aerial vehicles (UAV)

## Abstract

**Objectives:**

Unmanned Combat aerial vehicles (UAVs) have evolved into assets of tactical-critical importance, thus safeguarding the occupational health of their operators is imperative. As UAV units of the Chinese are predominantly stationed in high-altitude regions, this study aimed to systematically assess the occupational health status and risk factors faced by these operators during their service, and to propose targeted protective measures.

**Methods:**

A cohort of 62 active-duty UAV operators stationed in high-altitude regions was recruited to complete a self-administered questionnaire. The investigation was conducted utilizing a comprehensive framework encompassing occupational health assessment, risk factor evaluation, and protective measures analysis. Statistical methodologies employed included descriptive statistical analysis, symptom co-occurrence network analysis, and clustering pattern analysis.

**Results:**

UAV operators in high-altitude regions exhibited significantly higher prevalence of neurological (67.74%), musculoskeletal (64.52%), and psychological symptoms (46.77%) compared to their counterparts in plain areas, with additional manifestations of otorhinolaryngological (67.74%) and respiratory symptoms (64.52%). Symptom co-occurrence network analysis identified “musculoskeletal pain” and “memory impairment “as central hubs. Clustering analysis revealed distinct risk stratification, with high-, medium-, and low-risk subgroups constituting 20.97, 40.32, and 38.71% of the cohort, respectively. Among risk factors, “unreasonable work schedules” and “high-altitude hypoxia” received the two highest risk scores. Regarding protective measures, “health education,” (0.66) “scientific training protocols,” (0.62) and “rational shift scheduling” (0.60) demonstrated the highest comprehensive effectiveness scores.

**Conclusion:**

The UAV operator cohort in high-altitude regions demonstrated a notably poor occupational health status. “musculoskeletal pain” and “memory impairment “were identified as critical intervention targets. Given the prominent roles of “high-altitude hypoxia” and “unreasonable work-rest schedules” as dominant risk factors, occupational health strategies should be strategically redirected from over-reliance on personal protective equipment toward prioritized investment in efficient management systems. These include implementing “scientific training protocols,” enhancing “health education,” and establishing structured “work-rest rotation systems.” Simultaneously, essential oxygen supply and noise reduction equipment should be deployed at high-altitude workplaces.

## Graphical abstract

**Figure:**
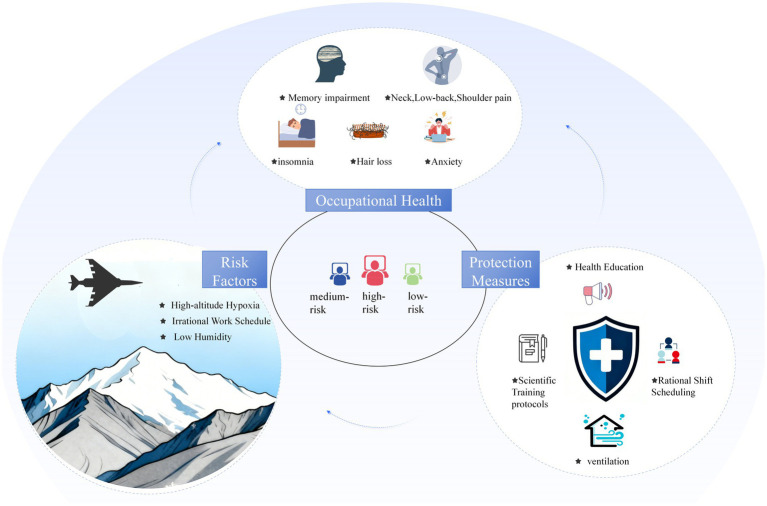

## 1 Introduction

Since the outbreak of the Russia-Ukraine conflict, reconnaissance-strike integrated UAVs have provided frontline units with beyond-visual-range strike capabilities, thereby reducing dependence on traditional fire support. Consequently, large military UAVs have emerged as tactically critical assets in modern warfare (1, 2). These systems can perform prolonged intelligence, surveillance, and reconnaissance (ISR) missions, delivering essential intelligence for operational decision-making, while simultaneously conducting remote strikes against enemy targets to effectively minimize personnel casualties and operational costs. As UAV operators have become indispensable components of military forces, they play increasingly vital roles in contemporary combat environments.

Compared to manned aircraft pilots, UAV operators demonstrate significantly higher prevalence rates of neurological and psychological symptoms (3). A study focusing on operators of armed UAVs, specifically the “Reaper” and “Global Hawk” platforms, revealed that approximately 46–48%% of pilots experienced severe psychological manifestations, including inattention, anxiety, and sleep disturbances (4). Furthermore, Grindley’s analysis of human factors in UAV accident reports demonstrated that among 72 medium-to-large UAV accidents occurring over the decade preceding 2022, 39 incident reports were attributed to human factors, accounting for 54.2% of all cases (5). Consequently, enhancing the occupational health of UAV operators represents a critical measure for preventing aviation accidents and ensuring overall flight safety (6).

Previous studies have established that the occupational health of UAV operators is predominantly influenced by their work environment (7–9). Moreover, U.S. military reports on MQ-1 Predator and MQ-9 Reaper operations identify characteristic occupational stressors including prolonged duty hours, shift work patterns, workstations with suboptimal ergonomic design, remote deployment locations, and real-time exposure to imagery depicting destruction and casualty scenarios, which have been demonstrated to contribute to elevated psychological distress among operators (10). These preliminary reports indicate that sustained operation under conditions of high workload intensity, extended working hours, and personnel shortages may potentially inflict greater physiological and psychological harm on UAV operators than exposure to combat imagery alone.

Furthermore, UAV Unit face additional health challenges associated with high-altitude environments, potentially posing more severe health risks compared to their plain-deployed counterparts. These regions are characterized by extreme environmental conditions including intense ultraviolet radiation, hypobaric hypoxia, significant atmospheric pressure fluctuations, strong winds, dramatic diurnal temperature variations, and extreme aridity. Consequently, occupational health concerns are particularly pronounced in these specialized working environments. Research by Alcantara-Zapata demonstrates that high-altitude exposure significantly influences individual stress levels (11). Environmental factors such as hypobaric hypoxia can trigger heightened depression and anxiety. Among high-altitude populations, including Tibetan natives, mountaineers, and military personnel, ocular vulnerability represents a particular health concern (12). Prolonged high-altitude exposure may impair sleep quality and physical performance among military staff, leading to accelerated fatigue during mission execution (13). Notably, the occupational burnout prevalence among military personnel stationed in high-altitude regions reaches (13)%, substantially exceeding rates observed in other occupational groups (13).

Hence, it is imperative to understand the occupational health implications of unique risk factors encountered by UAV operators during their service in high-altitude regions. Despite substantial research on aviation personnel health, studies specifically addressing the occupational health of high-altitude UAV operators remain scarce. Current efficacy assessments rely primarily on fragmented internal feedback, verbal reports, or informal communications with frontline commanders and operators. Furthermore, existing health investigations involving high-altitude military personnel typically evaluate physiological and psychological wellbeing in isolation, while largely overlooking the critical importance of systematic risk factor analysis and protective measure implementation.

To address this research gap, this study integrates three critical dimensions: occupational health symptoms, risk factors, and protective measures for UAV operators. Through the administration of on-site questionnaires to collect comprehensive data, we specifically focus on operators’ firsthand experiences to systematically analyze the health challenges and support needs faced by high-altitude UAV operators. The findings aim to inform the development of tailored occupational health interventions for this specialized military population.

This study selected UAV operators stationed in high-altitude regions as the research cohort. Building upon existing research in this field, we systematically investigated their occupational health symptoms, work-related risk factors, and the effectiveness of current protective measures. The collected data will provide crucial references for military commanders and healthcare management to better comprehend and address the unique challenges confronting UAV units in high-altitude environments. Our investigation encompasses four key dimensions: basic demographic characteristics, occupational health symptoms, occupational risk factors, and the utilization and efficacy of protective measures.

- Demographic characteristics (e.g., age, gender, marital status) and health behavior patterns (smoking history, alcohol consumption frequency);
- Occurrence and severity of discomfort symptoms across multiple systems: neurological, respiratory, otolaryngological, digestive, psychological, endocrine, dermatological, cardiovascular, urinary, and musculoskeletal;
- Health impacts of occupational risk factors, including physical, organizational/workload-related, biological, radiological, hazardous gases, and chemical exposures;
- Perceived effectiveness of protective strategies, categorized as administrative controls, engineering controls, and personal protective equipment (PPE).

## 2 Methods

### 2.1 Study participants

A purposive sampling method was employed to recruit UAV operators from a specific military unit stationed in a high-altitude region. Participants who were unavailable during the investigation period or provided incomplete survey information were excluded. To ensure representation across all subgroups of UAV operators, a stratified sampling approach was adopted based on age and rank. Consequently, questionnaires were distributed to and collected from 62 eligible participants. Prior to the interview, all individuals provided written informed consent.

This study was approved by the Ethics Committee of the Air Force Medical Center (Approval No.: AFMC (Research) No. 2025-57-PJ01). All procedures strictly adhered to established ethical guidelines and governance requirements. Researchers explicitly informed each participant that involvement was entirely voluntary, that they could withdraw at any time without penalty, and that no adverse consequences would result from participation.

### 2.2 Questionnaire design

The self-administered questionnaire was developed with inspiration from NASA’s “Integration of Unmanned Aircraft Systems into the National Airspace System Project” and Princeton University’s Indoor Safety Guidelines (3, 14, 15), and was further refined through expert interviews to align with the research objective of assessing and improving the occupational health of UAV operators in high-altitude areas. It comprised three sections: occupational health symptoms (66 items), workplace risk factors (47 items), and evaluations of the effectiveness of protective measures (26 items). Symptom severity was standardized using a four-point Likert scale (0 = none, 1 = mild, 2 = moderate, 3 = severe), with clearly defined mappings for numerical and categorical responses. Occupational risk factors were dichotomously scored (0 = absent, 1 = present), and protective measures were assessed using a standardized coding scheme (0 = not used, 1 = poor effectiveness, 2 = moderate effectiveness, 3 = high effectiveness).

### 2.3 Statistical analysis

#### 2.3.1 Descriptive analysis

Descriptive statistics were used to summarize participants’ demographic characteristics, including age, gender, marital status, educational level, smoking history, and alcohol consumption history.

#### 2.3.2 Assessment of occupational health symptoms

This assessment was grounded in the occupational health framework established in the U.S. Air Force Research Laboratory report “Reassessment of Occupational Health Among U.S. Air Force Remotely Piloted Aircraft Operators” (711th Human Performance Wing), with additional inputs derived from structured expert discussions (2). This system encompasses symptoms across 10 physiological domains. The complete governing equations and numerical implementation details are provided in the Supplementary Material (Supplementary Section S1).

1. The prevalence by organ symptom ( ${\text{Prev}}_{S}$ ) was calculated.
2. The Prevalence rate for the i -th symptom ( $P_{i}$ ) was defined as the proportion of individuals in the study population reporting that symptom.
3. The Severity Score for a specific symptom j ( $\bar{S}j$ ) (where *j* = 1, 2, …, m) was calculated
4. The total symptom burden for the k -th ( $\mathrm{TS}B_{k}$ ) individual was computed as the sum of the severity scores across all assessed symptoms.
5. Symptom network analysis was performed by constructing a symptom co-occurrence network, in which nodes represented symptoms (sized according to their frequency of occurrence) and edges reflected co-occurrence relationships between symptoms (weighted by their co-occurrence frequency). A spring layout was applied for network visualization, and only the top 30% of edges by co-occurrence frequency were included in the analysis.

#### 2.3.3 Assessment of occupational health risk factors

A risk factor assessment Framework was developed through a systematic literature review and structured expert discussions (2). The complete governing equations and numerical implementation details are provided in the Supplementary Material (Supplementary Section S2).

1. The prevalence of Exposure (EP) to a specific risk factor within the study population was calculated.
2. The Effect Size (ES) for each risk factor was calculated as the difference in the mean TSB between the exposed and unexposed groups, divided by the pooled standard deviation.
3. Breadth of Affected Physiological Systems (BAPS)

This metric represents the number of distinct body systems significantly affected by the risk factor, identified through Mann–Whitney U tests (significance level set at *p* < 0.05).

1. The Composite Importance Score (CRS)was then derived as a weighted sum, this weighting scheme prioritizes ES (50%), followed by BAPS (30%) and EP (20%), to identify key targets for intervention.

#### 2.3.4 Assessment of protective measures effectiveness

A similar multidimensional approach was employed to evaluate the effectiveness of protective measures. The complete governing equations and numerical implementation details are provided in the Supplementary Material (Supplementary Section S3).

1. The implementation rate (IR) for each protective measure was calculated.
2. The mean protective effectiveness (MPE) was calculated
3. The Composite Protective Effectiveness (CPE) was determined using a weighted evaluation approach that integrated both IR and MPE.

#### 2.3.5 Symptom pattern clustering

For symptom pattern clustering, system scores were first standardized using Z-scores. K-means clustering was then applied with a fixed random seed of 42 and 10 iterations. The optimal number of clusters was determined using the elbow method, and the characteristics of each resulting cluster were subsequently analyzed.

#### 2.3.6 Reliability and *post-hoc* power analysis

The internal consistency reliability of the multi-system symptom scales was assessed using Cronbach’s alpha coefficient. A *post-hoc* power analysis was performed. Given the observed sample size (*N* = 62) and an alpha level of 0.05, we calculated the achieved power to detect a medium effect size (Cohen’s *d* = 0.5) for a two-sample *t*-test under various group allocation scenarios.

All statistical analyses were performed using Python’s statsmodels library within the PyCharm software environment, with statistical significance set at *p* < 0.05.

## 3 Results

### 3.1 Baseline information for participants

A total of 62 questionnaires were distributed in this study, with 62 completed questionnaires returned, resulting in both a response rate and valid response rate of 100%. The demographic characteristics of the participants are summarized in Table 1, 100% of the participants were male, 9.7% held associate degrees, 88.7%% held bachelor’s degrees, and 1.6% held master’s degrees. Additionally, 48.3% reported a history of smoking, while 37.1% reported a history of alcohol consumption. Subsequent analysis confirmed that military status did not exert a significant influence on occupational health outcomes or any of its dimensions; therefore, related descriptions have been streamlined accordingly.

**Table 1:** Demographic characteristics of the study participants.

Characteristic	Subcategory	*n*
Age (years)	<25	16 (25.81%)
	26–30	23 (37.10%)
	31–35	8 (12.90%)
	>35	15 (24.19%)
Gender	Male	62
Marital status	Married	34 (54.8%)
	Other	28 (45.2%)
Educational level	Associate degree	6 (9.7%)
	Bachelor degree	55 (88.7%)
	Master or PhD	1 (1.6%)
Smoking	No	33 (53.2%)
	Yes	29(46.8%)
Drinking	No	39 (62.9%)
	Yes	23 (37.1%)

### 3.2 Occupational health symptoms

**Figure 1:**
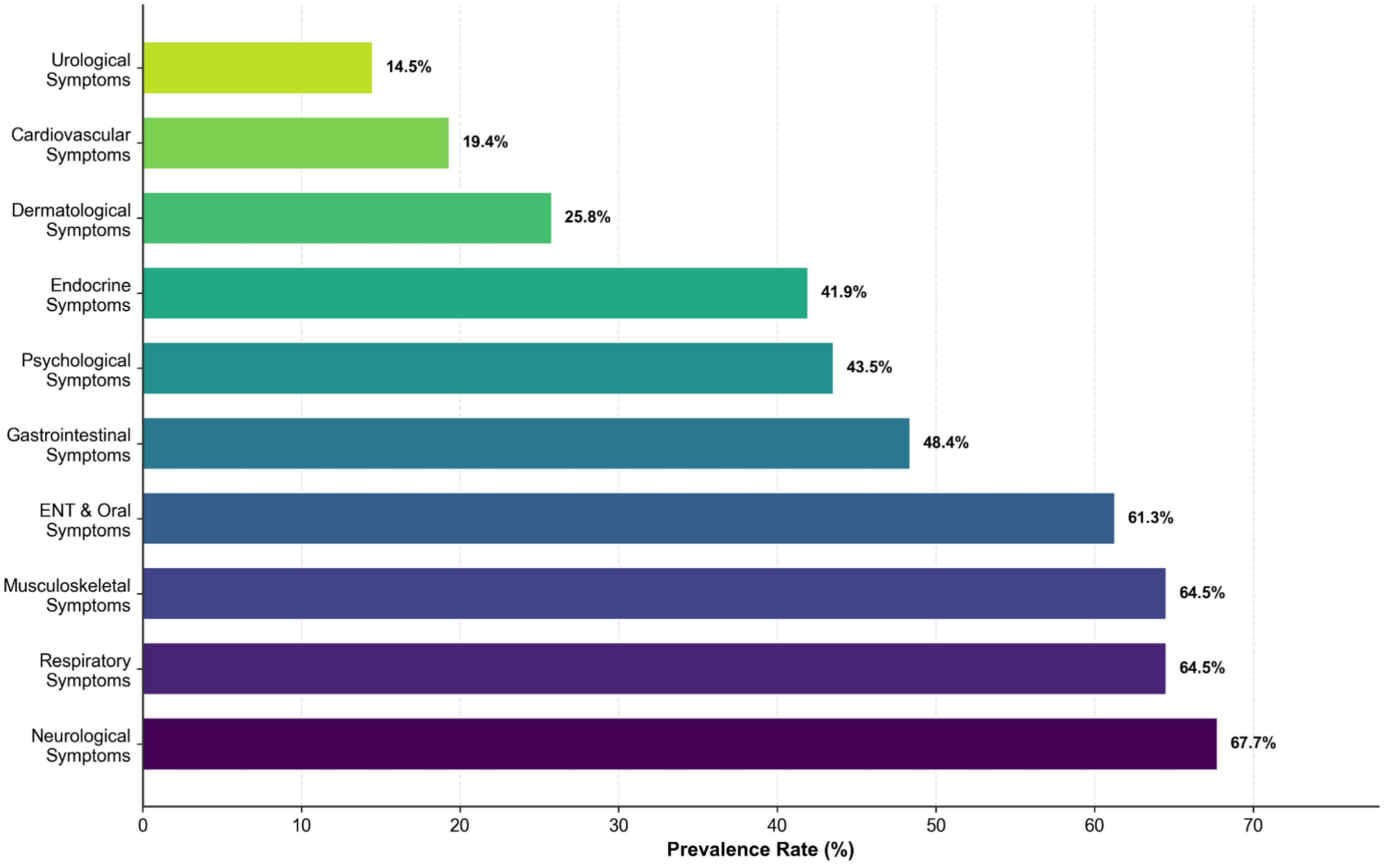
Prevalence rate by organ system. Neurological symptoms were the most common (67.7%), followed by musculoskeletal and respiratory symptoms (both 64.5%). Urinary system symptoms exhibited the lowest prevalence (14.5%). Colors gradient from light green (representing the lowest prevalence) to deep purple (representing the highest prevalence).

The ${\text{Prev}}_{S}$ among UAV operators is illustrated in Figure 1. The Prevalence by organ system, in descending order, were as follows: Neurological Symptoms (67.7%), Musculoskeletal Symptoms (64.5%), Respiratory Symptoms (64.5%), Otorhinolaryngological and Oral (ENT&Oral) Symptoms (61.3%), Gastrointestinal Symptoms (48.4%), Psychological Symptoms (43.5%), Endocrine Symptoms (41.9%), Dermatological Symptoms (25.8%), Cardiovascular Symptoms (19.4%), and Urological Symptoms (14.5%).

**Figure 2:**
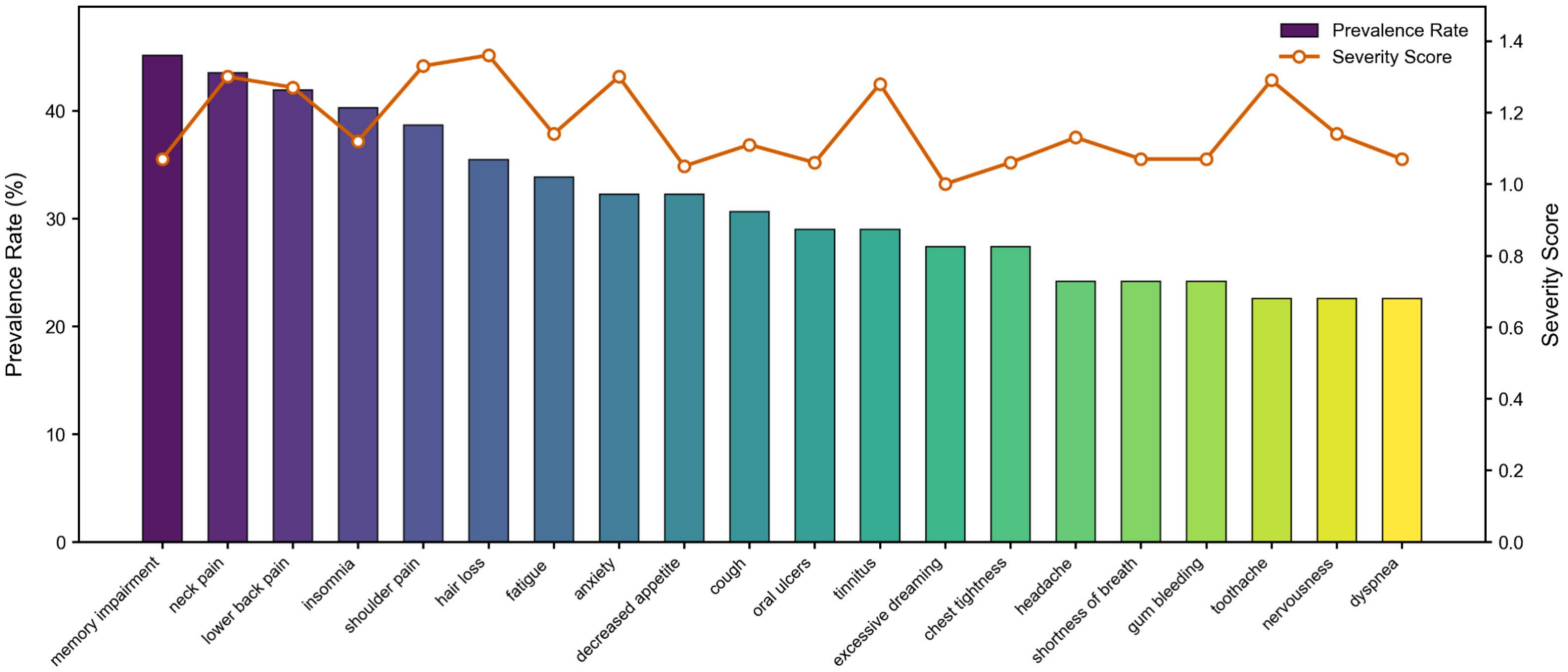
Prevalence and severity score of symptom. Memory impairment was the most common symptom, followed by neck pain, low back pain, and insomnia. The left vertical axis (*y*-axis) represents the prevalence (%) of each reported symptom, with colors ranging from purple (highest values) to yellow (lowest values). The right vertical axis indicates the severity scores for the corresponding symptoms, depicted by a red line with circular markers. Symptoms on the *x*-axis are arranged in descending order of prevalence.

The top 20 symptoms were ranked in descending order of prevalence rates, with their severity scores calculated, as shown in Figure 2. The most prevalent symptoms, in descending order, were: memory impairment (prevalence 45.2%, severity score 1), neck pain (43.5%, 1.3), lower back pain (41.9%, severity score 1.3), insomnia (40.3%, 1.1), shoulder pain (38.7%, 1.3), hair loss (35.5, 1.4), fatigue (33.9%, 1.1), anxiety (32.3%, 1.3), decreased appetite (32.3%, 1.1), cough (30.6%, 1.1), oral ulcers (39.0%, 1.1), tinnitus (29.0%, 1.3), excessive dreaming (27.4%, 1.0), chest tightness (27.4%, 1.1), headache (24.2%, 1.1), shortness of breath (24.2%, 1.1), gum bleeding (24.2%, 1.1), nervousness (22.6%, 1.1), toothache (22.6%,1.3), and dyspnea (22.6%, 1.1).

**Figure 3:**
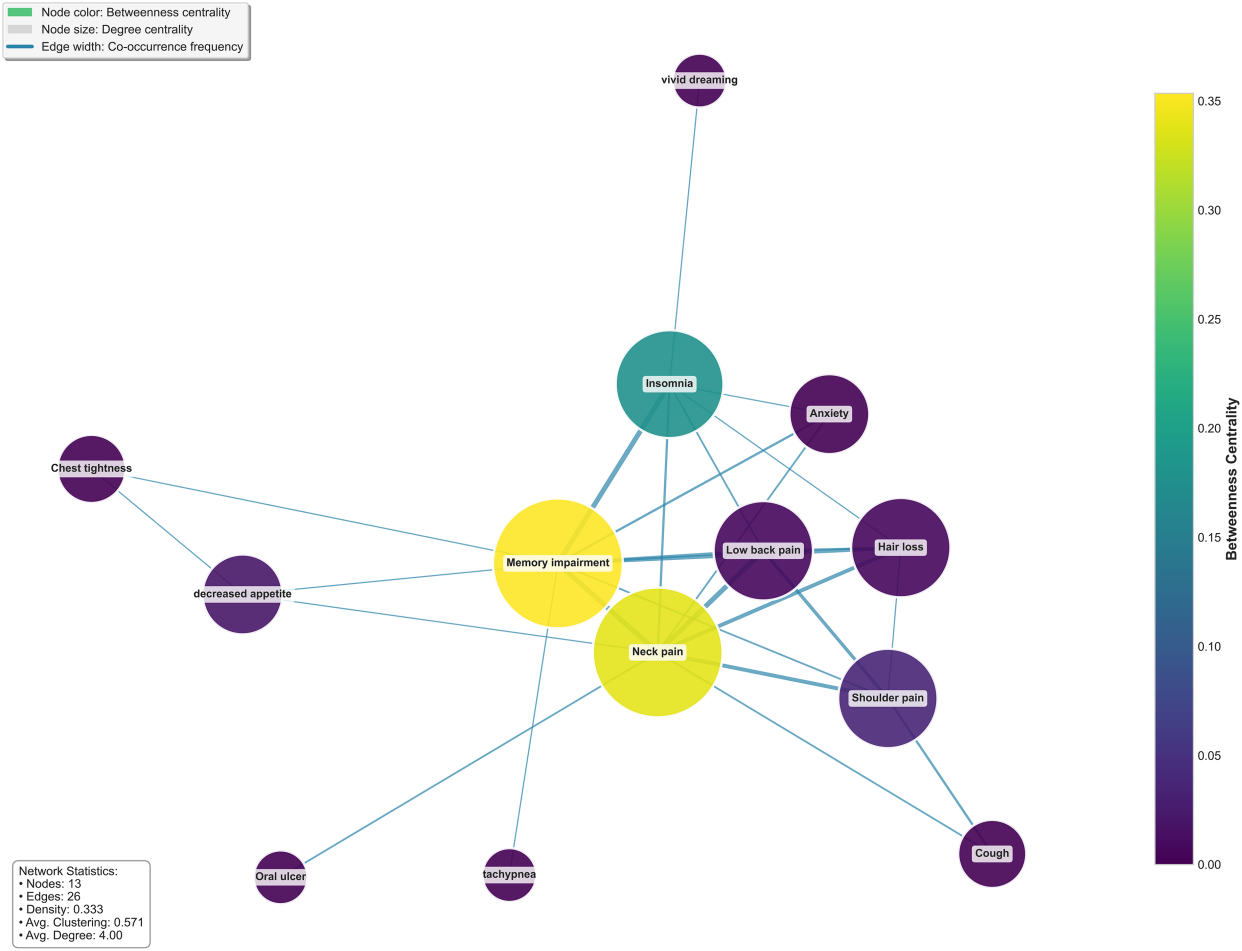
Symptom co-occurrence network among UAV operators in plateau regions. Memory impairment and neck pain were identified as central hub symptoms. Nodes represent individual symptoms, with node size corresponding to degree centrality (number of direct connections to other symptoms) and node color reflecting betweenness centrality [ranging from yellow (highest) to purple (lowest)]. Edge width is proportional to the co-occurrence frequency of each symptom pair. Network metrics: 13 nodes, 26 edges, network density = 0.333, average clustering coefficient = 0.571, average degree = 4.00.

Given the potentially complex interrelationships among multidimensional symptoms in high-altitude environments, which are difficult to disentangle using traditional analytical approaches, we conducted a symptom network analysis to elucidate their underlying structure. Symptom co-occurrence network analysis revealed interconnected relationships among multi-system symptoms in the high-altitude UAV operator cohort, forming a network topology with the neuro-psychological-musculoskeletal system as its core structure (Figure 3). The analysis identified several key symptom nodes and their strong interconnections. “neck pain” and “memory impairment” constituted the most prominent connection hubs, forming tight clusters with “insomnia “and “anxiety.” This indicates that “neck pain” and “memory impairment “represent core occupational health concerns, demonstrating significant co-occurrence patterns with cognitive decline, emotional distress, and somatic pain. Musculoskeletal symptoms exhibited distinct clustering, with strong connections observed among “neck pain “, “lower back pain,” and “shoulder pain.”

### 3.3 Occupational health risk factors

#### 3.3.1 Prevalence of exposure

**Figure 4:**
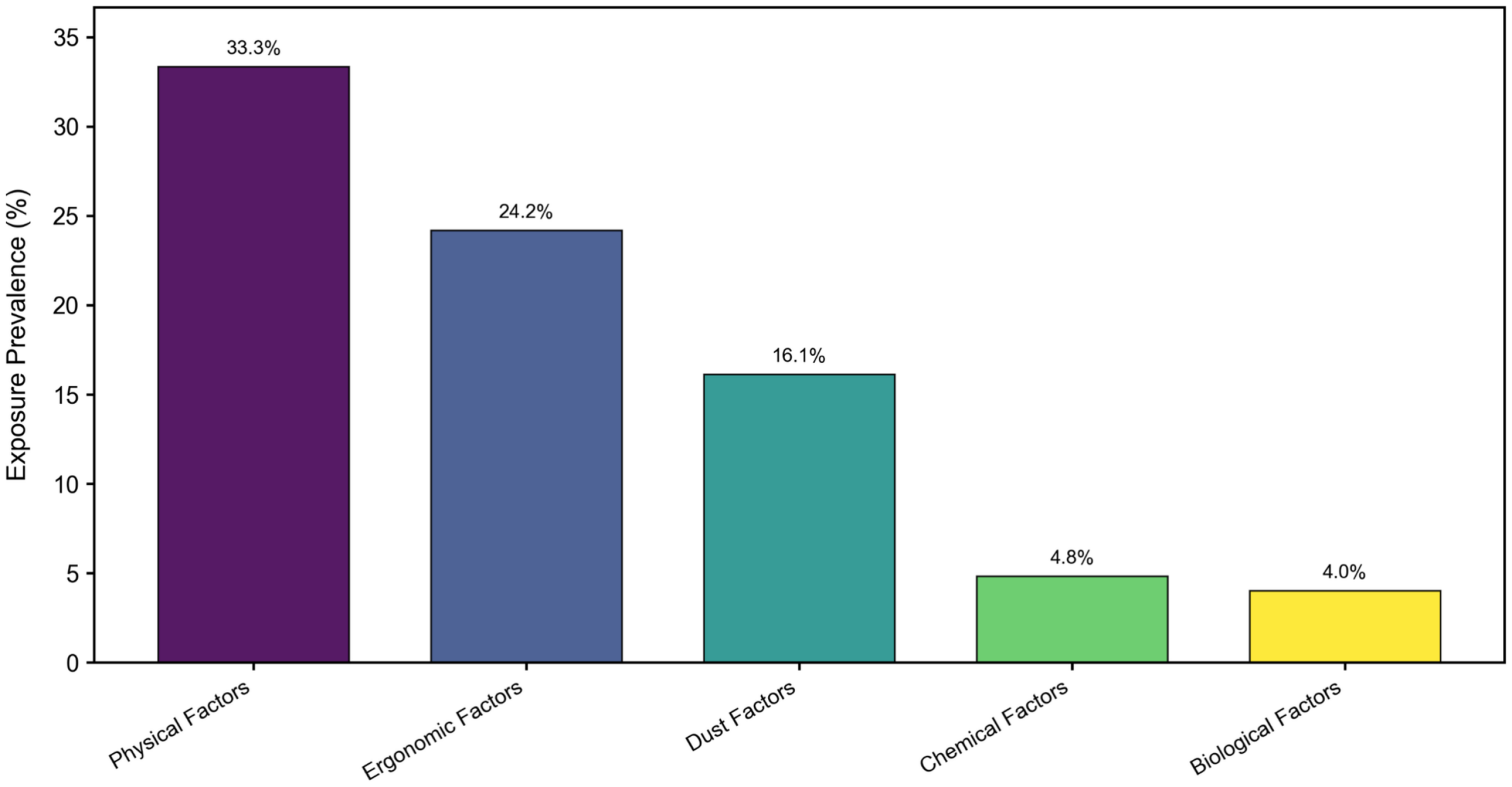
Prevalence rate of exposure. Physical factors were the most prevalent, followed by ergonomic factors. Biological factors exhibited the lowest exposure prevalence. Colors range from deep purple (representing the highest exposure prevalence) to yellow (representing the lowest exposure prevalence).

As illustrated in Figure 4, the survey results demonstrated that physical factors exhibited the highest average exposure prevalence (33.3%) among UAV operators in the high-altitude region. This was followed by ergonomic factors (24.2%), dust (16.1%), chemical factors (4.8%) and biological factors (4.0%).

#### 3.3.2 Association between risk factor exposure prevalence and effect size

**Figure 5:**
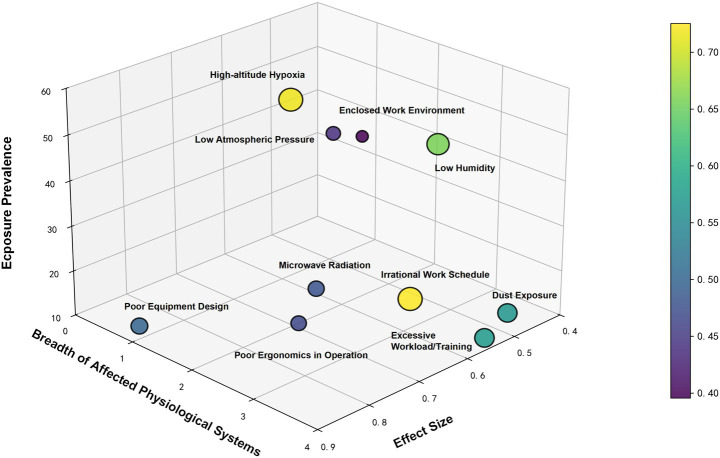
Three-dimensional visualization of risk factor based on exposure prevalence, systemic impact breadth, and effect size. The *x*-axis represents the effect size, the *y*-axis represents the systemic impact breadth, and the Z-axis represents exposure prevalence. The color gradient of the data points (from purple to yellow) quantifies the composite importance score of the hazard factors.

A scatter plot analysis was utilized to quantitatively evaluate the top 10 risk factors based on their exposure prevalence (x-axis), effect size (*y*-axis), and magnitude of effect (color-coded scale) (Figure 5). Among these factors, “high-altitude hypoxia” demonstrated the highest exposure prevalence (59.7%) with an effect size of 0.7, followed by “irregular work-rest schedules” (exposure prevalence: 29.03%; effect size: 0.72) and “low humidity” (exposure prevalence: 48.39%; effect size: 0.534). Other significant risk factors included “poor equipment design” (12.9%; 0.886), “inadequate illumination” (16.12%; 0.662), “excessive workload/training” (12.9%; 0.57), “dust exposure” (16.13%; 0.52), “microwave radiation” (16.13%; 0.652), “awkward postures/movements” (9.68%; 0.69), and “low atmospheric pressure” (38.71%; 0.49).

This study employed a multidimensional evaluation framework to systematically assess the health impacts of various risk factors in the occupational environment. The Composite Risk Score revealed that the top 15 risk factors had scores ranging from 0.266 to 0.725, with “irrational work schedule” ranking first with a composite score of 0.725, highlighting its predominant role in occupational health risks (Table 2).

**Table 2:** Composite importance score of risk factors.

No.	Risk factors	Composite importance score
1	Irrational work schedule	0.725
2	High-altitude hypoxia	0.717
3	Low humidity	0.658
4	Excessive workload/training	0.573
5	Dust exposure	0.567
6	Poor equipment design	0.499
7	Microwave radiation	0.48
8	Poor ergonomics in operation	0.466
9	Low atmospheric pressure	0.440
10	Enclosed work environment	0.396

The risk factors demonstrated the following distribution characteristics: (1) Physical factors exerted comprehensive influences, with physical factors being particularly prominent in the rankings (7/15, 46.7%). These encompassed various plateau-specific environmental conditions including “high-altitude hypoxia” (0.717), “low humidity” (0.658), “ultraviolet radiation” (0.315), “high temperature” (0.384), and “low atmospheric pressure” (0.440). (2) Ergonomic factors played a dominant role, accounting for a significant proportion (6/15, 40%) among the top 15 risk factors. In addition to the top-ranked “irrational work schedule,” factors such as “excessive workload/training” (0.573), “Poor Ergonomics in Operation “(0.460),"poor equipment design” (0.499), and Enclosed Work Environment (0.396) demonstrated high importance scores. (3) “Dust exposure,” as the sole independently classified factor, ranked in the middle range with a composite score of 0.567, indicating its unique health impact in specific occupational environments. (4) Various electromagnetic radiation factors (“ultra-high frequency electromagnetic fields” 0.36, “microwave radiation” 0.48) all presented certain health risks.

### 3.4 Protective measures

#### 3.4.1 Implementation rate and average protective effectiveness

**Figure 6:**
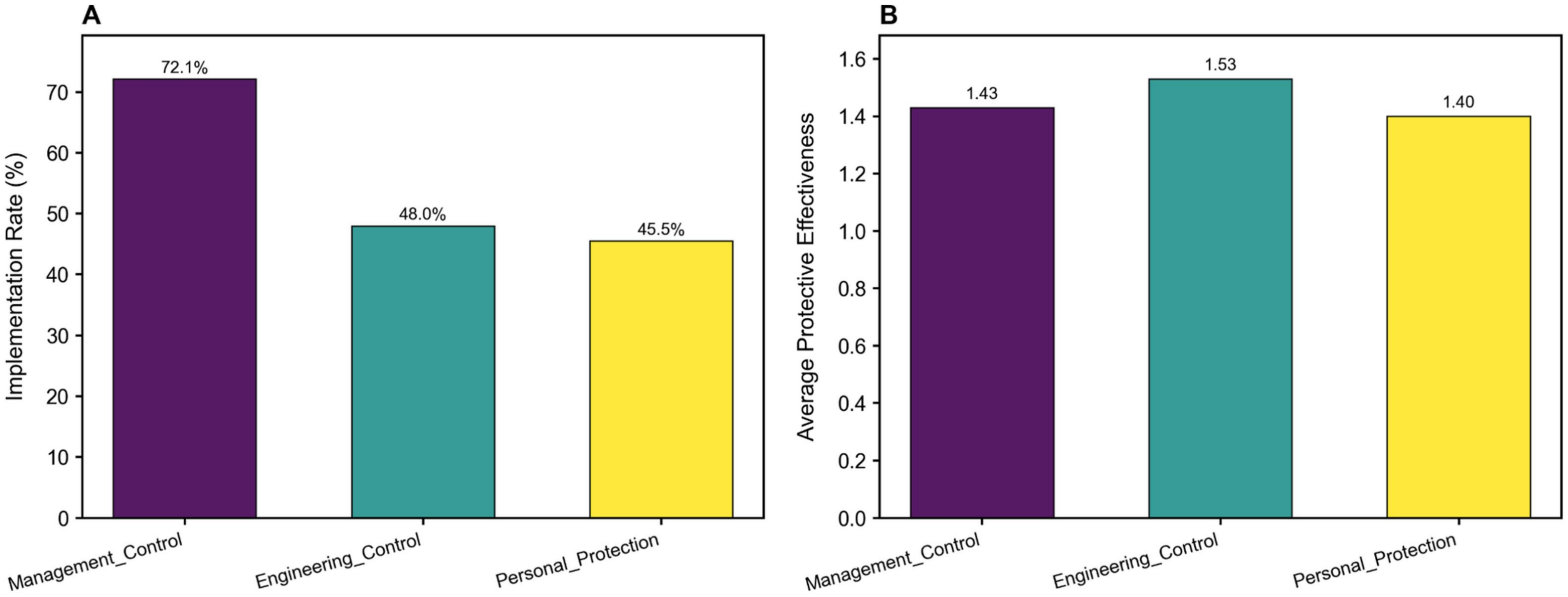
Comparison of implementation rate and average protective effectiveness across different protective measures. **(A)** Shows the implementation rates of the three strategies, with management control having the highest rate, considerably higher than engineering control and personal protective measures. **(B)** Displays the mean protection effectiveness of the three strategies. Engineering controls exhibited the highest mean protection effectiveness, followed by management controls and personal protective measures.

As shown in Figure 6, management controls demonstrated an implementation rate of 71.2% with a protective effectiveness of 0.567, followed by engineering controls (implementation rate: 48.0%; effectiveness: 0.498) and personal protective equipment (implementation rate: 42.1%; effectiveness: 0.462).

#### 3.4.2 Composite protective effectiveness

Based on the comprehensive effectiveness assessment formula, the occupational health protection measures were systematically ranked. Among the top 10 ranked measures (Table 3), management control measures demonstrated significant advantages, dominating the rankings. Specifically, “health education” achieved the highest composite score (0.66), while “scientific training protocols” (0.62), “rational shift scheduling” (0.60), “hazard protection education” (0.58), and “publicity and enforcement of health standards” (0.53) also showed high protective effectiveness. Additionally, engineering controls including “ventilation” (0.59), “noise reduction” (0.56), and “air purification” (0.52), along with the personal protective measure “regular health examinations” were ranked among the most effective interventions, collectively forming the most effective cluster of protective measures.

**Table 3:** Composite protective effectiveness of protective measures.

Protective measure category	Protective measures	Implementation rate (100%)	Mean protective effectiveness (MPE)	Composite protective effectiveness
Management control	Health education	90.32	1.5	0.66
Management control	Scientific training protocols	82.26	1.45	0.62
Management control	Rational shift scheduling	83.87	1.31	0.60
Engineering controls	Ventilation	79.03	1.35	0.59
Management control	Hazard protection education	74.19	1.43	0.58
Personal protective equipment	Regular health examinations	77.42	1.31	0.57
Engineering	Noise reduction	46.77	1.86	0.56
Management control	Publicity and enforcement of health standards	66.13	1.34	0.53
Management control	Monitoring of influencing factors	53.23	1.58	0.53
Engineering	Air purification	46.77	1.66	0.52

### 3.5 Risk stratification based on cluster analysis

**Figure 7:**
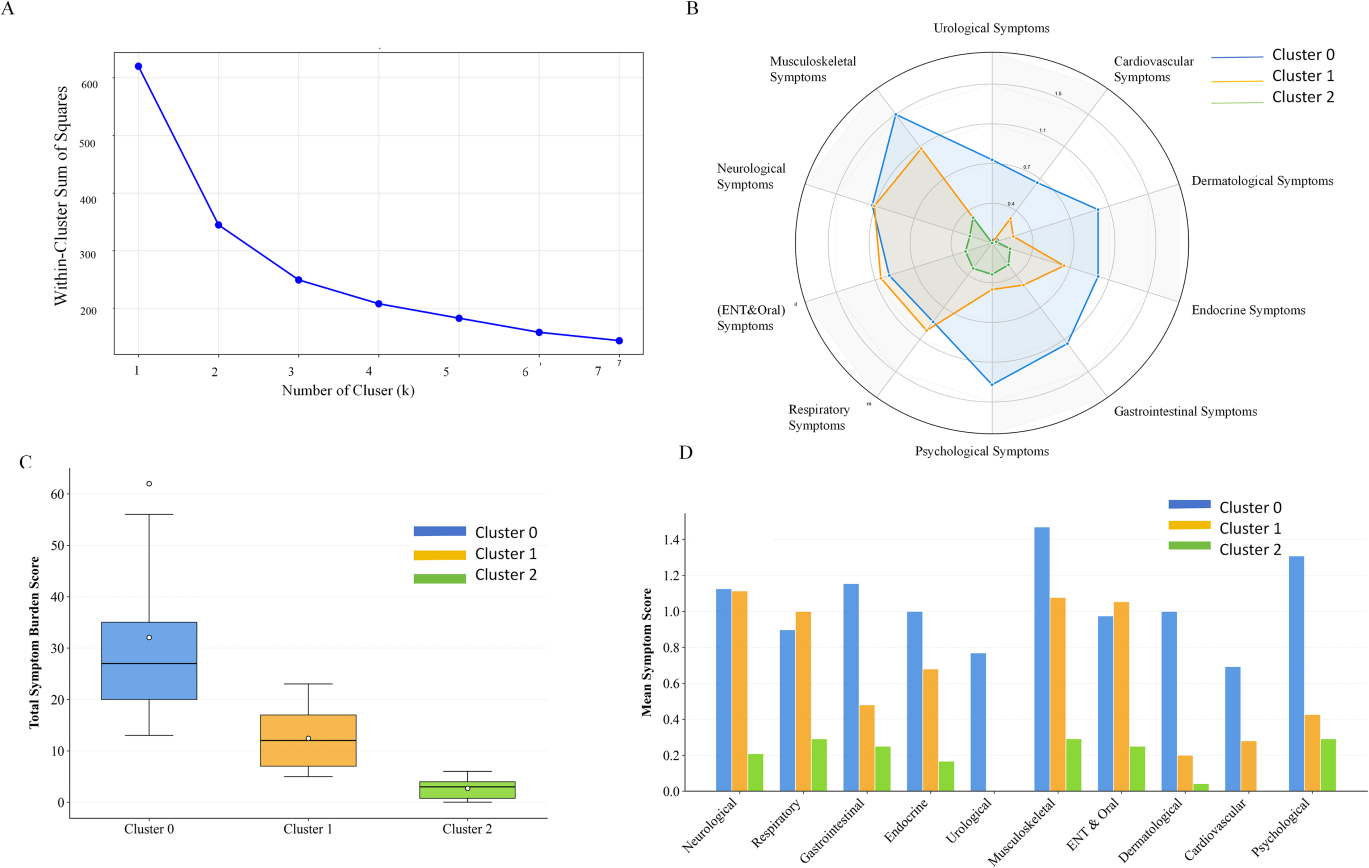
Three distinct subpopulations of high-altitude UAV operators. **(A)** Elbow method plot indicating the optimal number of clusters (*k* = 3), suggesting that partitioning the population into three subgroups is most appropriate. **(B)** Average symptom score profiles of cluster 0, cluster 1, and cluster 2 across distinct symptom domains. **(C)** Comparison of mean total symptom burden scores among the three clusters: cluster 0 = 32.08, cluster 1 = 12.44, and cluster 2 = 2.71. **(D)** Detailed presentation of mean symptom scores for each cluster across the nine specific symptom domains. In **(B–D)**, blue represents cluster 0, orange represents cluster 1, and green represents cluster 2.

To identify potential subgroups with distinct health risk profiles, an unsupervised clustering analysis was conducted. The optimal number of clusters for occupational health symptom patterns was determined using the elbow method, followed by the application of the K-means algorithm. This data-driven approach aims to provide insights for developing targeted interventions. As shown in Figure 7A, the Within-Cluster Sum of Squares (WCSS) decreased monotonically with increasing cluster numbers (k), consistent with expectations. The WCSS demonstrated a pronounced reduction from k = 1 (620) to k = 2 (347.6). A substantial decrease in WCSS (ΔWCSS = 67.82) persisted when the number of clusters increased from 2 to 3. However, beyond k = 3, the WCSS decline curve exhibited a distinct flattening trend, indicating substantially diminished marginal gain. Specifically, the reduction in WCSS (ΔWCSS) from k = 3 to k = 4 and from k = 4 to k = 5 was considerably smaller than the decrease observed from k = 2 to k = 3. Consequently, k = 3 was identified as the “elbow point” of the curve, suggesting limited improvement in model fit with additional clusters beyond this point. Based on this analysis, k = 3 was selected as the optimal number of clusters for subsequent symptom pattern clustering analysis.

Based on K-means clustering analysis (k = 3), this study successfully identified three distinct subpopulations of high-altitude UAV operators with significantly different symptom profiles (Figures 7B–D) which delineates the characteristics of each subgroup.

Cluster 0 (High-Risk Group, *n* = 13, 20.97%): This subgroup demonstrated substantially higher symptom scores across all 10 physiological systems compared to the other two clusters, forming a complete, large-area radar profile. With an average total symptom burden of 32.08, they exhibited particularly severe symptoms in the musculoskeletal and psychological systems, alongside notable manifestations in the integumentary, nervous, and digestive systems. This pattern indicates that this small subpopulation experiences comprehensive, multi-system health pressures.

Cluster 1 (Moderate-Risk Group, *n* = 25, 40.32%): This cluster exhibited an intermediate symptom profile between Cluster 0 and Cluster 1, with an average total symptom burden of 12.44 and a medium-sized radar chart. They maintained relatively elevated scores in the nervous, psychological, respiratory, and musculoskeletal systems, representing a subpopulation experiencing moderate health impacts primarily centered on neuro-psychological-respiratory-musculoskeletal symptoms.

Cluster 2 (Low-Risk Group, *n* = 24, 38.71%): Characterized by the lowest symptom scores across all systems and a minimal average total symptom burden of 2.71, this cluster displayed the smallest and relatively flat radar profile. This subgroup represents the healthiest portion of the study population, reporting overall mild symptom burden.

### 3.6 Reliability and *post-hoc* power analysis results

The internal consistency of the symptom scales across ten physiological systems ranged from acceptable to good. As shown in Table 4, Cronbach‘s *α* coefficients were above 0.70 for seven out of 10 systems. The *post-hoc* power analysis revealed that the statistical power to detect a medium effect size (*d* = 0.5) was 0.85 under balanced group allocation (31 vs. 30), but dropped to 0.59 under an unbalanced allocation (40 vs. 21).

**Table 4:** Assessment of scale reliability: Cronbach’s alpha by physiological system.

Physiological system	Number of items	Cronbach’s α	95% CI	Reliability level
Neurological symptoms	9	0.828	[0.755, 0.886]	Good
Respiratory symptoms	7	0.779	[0.684, 0.854]	Acceptable
Gastrointestinal symptoms	6	0.685	[0.546, 0.793]	Adequate
Endocrine symptoms	4	0.494	[0.252, 0.672]	Poor
Urological symptoms	4	0.830	[0.749, 0.890]	Good
Musculoskeletal symptoms	13	0.777	[0.684, 0.851]	Acceptable
Otorhinolaryngological and oral (ENT&Oral) symptoms	7	0.545	[0.346, 0.700]	Poor
Dermatological symptoms	5	0.798	[0.706, 0.868]	Acceptable
Cardiovascular symptoms	3	0.700	[0.543, 0.810]	Acceptable
Psychological symptoms	4	0.648	[0.477, 0.773]	Adequate

## 4 Discussion

This study conducted a systematic analysis of occupational health symptoms among Unmanned Aerial Vehicle (UAV) operators in China’s high-altitude regions. By quantifying risk factors and evaluating the implementation and effectiveness of protective measures, the findings indicate a generally poor state of occupational health within this cohort. The factors “neck pain “and “memory impairment “were identified as core intervention targets for priority management. Furthermore, “high-altitude hypoxia” and “irregular work-rest schedule” constitute significant risk factors. The focus of protective strategies should shift from an over-reliance on personal protective equipment towards prioritizing limited resources for the establishment of efficient management systems, such as “health education” “scientific training protocols “and “Rational Shift Scheduling “. Simultaneously, essential oxygen supply and noise reduction equipment should be deployed at high-altitude workplaces. This research addresses a critical gap in the occupational health assessment of this specialized population, providing a valuable foundation for understanding health risks in high-altitude occupational environments and formulating targeted protective interventions.

### 4.1 Occupational health

The UAV operator cohort stationed in high-altitude regions consisted exclusively of young and middle-aged males (aged 30.88 ± 2.76 years), with a married proportion reaching 54.8%. Occupational health symptoms demonstrated multi-system and multi-dimensional characteristics. Specifically, neurological (67.74%%), musculoskeletal (64.52%%), and psychological symptoms (46.77%) among these operators were more severe compared to those reported in previous studies of UAV operators in plain areas. Furthermore, pronounced otorhinolaryngological (67.74%) and respiratory symptoms (64.52%) were notably prevalent.

This study found that insomnia (severity score: 1.1) and memory impairment (severity score: 1) were reported by 40.3and 45.2% of UAV operators, respectively. These prevalence rates are significantly higher than the 22.8–35% range reported among operators in non-plateau regions such as the United States and Canada (16–18). While poor sleep quality and persistently elevated fatigue indices above critical thresholds are commonly reported among UAV operators in plain areas, high-altitude exposure is further associated with reduced sleep efficiency, diminished slow-wave sleep, and increased awakening indices (19). Musculoskeletal symptoms were particularly prominent in our high-altitude cohort, including neck pain (incidence: 43.5%, severity: 1.3), lower back pain (incidence: 41.9%, severity: 1.3), and shoulder pain (incidence: 38.7%, severity: 1.3). These incidences and severity levels exceeded the average rate of 37.38% reported for UAV operators in plain regions (2). Furthermore, psychological symptoms were more severe in the high-altitude group. The overall incidence of psychological symptoms was 46.77% in our study participants, with anxiety symptoms occurring in 32.3% of cases (severity: 1.3). This compares to the 14–33% prevalence of psychological symptoms reported among active and reserve Predator/Reaper UAV operators (20).

Symptom co-occurrence network analysis revealed the complex interplay of multi-system health impairments among UAV operators in high-altitude regions. The identification of “musculoskeletal Pain” and “Memory impairment” as central hubs within the network suggests these symptoms should be prioritized as core intervention targets. The emergence of a neuro-psycho-musculoskeletal symptom complex substantiates the psychosomatic interaction framework, wherein these central symptoms potentially influence each other and propagate to other systems through multiple pathways. Specifically, hypothalamic–pituitary–adrenal axis dysfunction may contribute to central fatigue, sleep disturbances, and heightened pain sensitivity (21), while autonomic nervous system dysregulation could mediate the relationship between psychological stress and various somatic symptoms (22, 23). Consequently, interventions targeting these central symptoms may yield beneficial effects across the entire symptom network.

The observed “shoulder pain-cough” linkage may be attributed to chronic cough inducing overload of respiratory accessory muscles (e.g., trapezius and sternocleidomastoid), potentially triggering myofascial pain syndrome (24, 25).

The overall occupational health status of UAV operators in high-altitude regions was notably poor, with only 38.71% classified as low-risk. The high-risk subgroup (20.97%) demonstrated a “multi-system health deterioration” profile, representing the priority target for intensive individualized interventions. The medium-risk subgroup (40.32%) exhibited the characteristic “neuro-psycho-musculoskeletal core” pattern, reflecting the most representative subhealth state in this population, consistent with the symptom co-occurrence network findings. Importantly, the existence of a low-risk subgroup demonstrates that maintaining relatively favorable health status remains achievable even under identical occupational exposures.

### 4.2 Risk factors

Compared to their counterparts in plain areas, UAV operators in high-altitude regions identified physical factors (33.3%) as significant occupational health risks, in addition to operational hazards. High-altitude hypoxia (exposure effect 0.72), recognized as the predominant physical stressor, received high-risk ratings reflecting its profound impact on physiological function—a finding consistent with previous meta-analyses on high-altitude exposure. Hypoxia contributes to health impairment through mechanisms including enhanced oxidative stress, disrupted energy metabolism, and neuroendocrine dysregulation. Chronic exposure to high altitudes induces structural and functional alterations in the motor cortex, manifested as reduced gray matter volume, diminished cerebrovascular reactivity, cortical thinning, and gradual decrease in cerebral blood flow (26–30). These changes are associated with pronounced decline in psychomotor function and long-term memory, alongside moderate deterioration in working memory and language performance (31, 32). Furthermore, characteristic high-altitude climate factors including intense ultraviolet radiation (0.315), low atmospheric pressure (0.440), low humidity (0.658), and temperature extremes (0.384) were also identified as high-risk factors, which collectively constitute the complex environmental exposure profile unique to high-altitude regions.

Notably, occupational factors constituted a substantial proportion of the top 15 risk factors identified, including “unreasonable work-rest schedules” (0.725), “excessive workload/training” (0.573), “poor ergonomic equipment design” (0.499), and “improper operating postures/movement patterns” (0.460). Specifically, irregular work-rest schedules and confined working environments were frequently associated with psychological symptoms such as irritability and depression, while suboptimal equipment design and sustained awkward postures contributed significantly to musculoskeletal pain. A sedentary work pattern was identified as a significant risk factor for neck pain (OR = 1.5) (33). The combination of chronic sleep deprivation and prolonged sedentary behavior among UAV operators represents a substantial risk profile for developing diabetes, cardiovascular diseases, and other metabolic disorders (29).

Furthermore, the interaction between occupational and environmental risk factors warrants particular attention. These factors may potentially exacerbate the health effects of environmental exposures through their impact on psychological stress levels and sleep quality.

Despite the unique high-altitude environment, traditional industrial hazards such as “dust exposure” (0.567) remained significant health concerns. Dust exposure adversely affects the respiratory system through mechanisms including alveolar epithelial cell damage, oxidative stress, and activation of inflammatory responses, ultimately contributing to respiratory symptoms.

UAVs maintain continuous communication with ground control stations through ultra-high frequency (UHF) or microwave band radio waves. Communication base stations and radar installations, commonly located near operator worksites, were identified as significant sources of electromagnetic radiation (EMR). In high-altitude environments, the thinner air may necessitate increased transmission power to maintain communication stability, potentially elevating operators’ exposure intensity. Our results indicated that various EMR exposures (UHF 0.36, microwave 0.48) posed measurable health risks. Furthermore, existing research demonstrates that chronic microwave exposure can lead to persistent cognitive impairment, accompanied by alterations in NMDA receptor subunits—a potential molecular mechanism underlying memory deficits (30).

### 4.3 Protective measures

The increasing prevalence of drone warfare in the 21st century, alongside the evolution of military operations, necessitates greater attention to occupational health protection measures for UAV operators.

Our findings, interpreted through the Hierarchy of Controls framework (34), provide evidence for optimizing protective strategies based on the “utilization-effectiveness” balance. Notably, five of the top 10 protective measures belonged to administrative controls, highlighting the crucial role of systematic, organizational-level interventions. “Health education” (0.66) and “hazard protection training” (0.58) enhance operators’ health literacy regarding high-altitude environmental risks, enabling them to actively acquire preventive knowledge regarding disease prevention, altitude adaptation, scientific work-rest scheduling, and proper use of protective equipment.

Furthermore, “scientific training protocols” (0.62) and “rational shift scheduling” (0.60) directly reduce exposure intensity and duration by fundamentally redesigning work processes. These measures specifically address the widely reported issues of “excessive workload” and “unreasonable work-rest schedules” that contribute to fatigue and insomnia. This approach aligns with the Hierarchy of Controls principle prioritizing engineering and administrative solutions over reliance on personal protective equipment (35). Importantly, shift work and weekly workloads exceeding 50 h demonstrate positive correlation with PTSD incidence in this population (36, 37).

“Regular physical examinations,” ranking sixth in comprehensive protective effect, demonstrated relatively high utilization rates (77.42%) but comparatively lower effectiveness (1.31%). In contrast, personal protective equipment (PPE) categories exhibited an inverse correlation between utilization and protective efficacy, with respiratory protection showing the lowest usage yet highest effectiveness. This pattern stems from the inherent passivity of PPE; although such equipment can effectively block hazards, factors including discomfort and usage costs frequently compromise compliance. These findings reinforce that PPE should be positioned as the last line of defense within the Hierarchy of Controls, implemented only after more effective intervention strategies.

Future efforts should prioritize cost-effective engineering controls (e.g., ventilation systems) and address implementation barriers for highly effective but underutilized measures (e.g., respiratory protection and cooling devices). Simultaneously, strengthening administrative controls will enhance systemic coordination. This integrated approach will maximize overall protective efficacy, ultimately establishing a comprehensive framework encompassing engineering, administrative, and personal protective measures.

## 5 Conclusion

This investigation revealed a notably poor health status among UAV operators in high-altitude regions, with only 38.71% identified as a low-risk population. “Musculoskeletal pain” and “Memory impairment” should be prioritized as core intervention targets for occupational health management in this cohort. The risk factors “high-altitude hypoxia” and “unreasonable work-rest schedules” were particularly prominent. Consequently, the focus of occupational health interventions should shift from an over-reliance on personal protective equipment towards redirecting limited resources to high-efficacy systemic measures. These include implementing “scientific training protocols,” enhancing “health education,” establishing scientific work-rest rotation systems, and deploying essential oxygen supply and noise reduction equipment and in high-altitude workplaces.

## 6 Limitations and future perspectives

This study has several limitations. First, the relatively limited sample size, attributable to the specialized nature of the population, necessitates validation in larger cohorts to confirm the stability of the findings. The primary limitation of this study is its modest sample size, which constrains statistical power. Our *post-hoc* analysis indicates that while the study had adequate power (0.85) for balanced comparisons, the power was insufficient for detecting medium effects in unbalanced comparisons. It should be noted that the participants of this study were exclusively recruited from UAV operators working in high-altitude regions within specific units. Consequently, the findings are primarily applicable to populations operating in similar environmental and mission-specific contexts. Generalization of the results to other groups requires further verification. Furthermore, while the reliability of most symptom scales was acceptable, the lower Cronbach’s alpha for the endocrine and otolaryngological scales suggests that findings related to these systems should be interpreted with caution. Future studies should conduct an *a priori* power analysis. For instance, to achieve 80% power for detecting a medium effect (*d* = 0.5), a total sample size of approximately 128 participants would be required.

## Data Availability

The raw data supporting the conclusions of this article will be made available by the authors, without undue reservation.
