# Supplementary material for: Occupational health, risk factors, and protection among unmanned aerial vehicle operator in the high-altitude region of China: an observational study
Source: Front Public Health. 2026 Apr 1;14:1764053. doi: 10.3389/fpubh.2026.1764053 (PMC13079638; doi:10.3389/fpubh.2026.1764053)
Supplement: Supplementary file 1 [file Supplementary_file_1.DOCX]

## Supplementary Material

## Section S1

1. Prevalence by Organ System($\text{Prev}_{S}$)

The prevalence of symptoms for each organ system S was calculated as:

$$\text{Prev}_{\mathbf{S}}\mathbf{=}\frac{\mathbf{N}_{\text{s}}}{\mathbf{N}}\boldsymbol{\times100\%}$$

*Where:*

$\text{Prev}_{S}$ = symptom prevalence (%) for system *S*.

$N_{S}$ = number of participants reporting at least one symptom in system *S*.

$N$ = total study population.

1. Symptom Prevalence ($P_{i}$)

The incidence rate for the $i$-th symptom was defined as the proportion of individuals in the study population reporting that symptom:

$$\boldsymbol{P}_{\boldsymbol{i}}\boldsymbol{=}\frac{\boldsymbol{N}_{\boldsymbol{i}}}{\boldsymbol{N}}\boldsymbol{\times100\%}$$

Where:

$N_{i}$ = the number of individuals reporting the $i$-th symptom.

(3) Severity Score for an Individual Symptom ($\overline{S}j$)

The Severity Score for a specific symptom *j* (where *j* = 1, 2, …, *m*) was calculated using the following equation:

$$\overline{\mathbf{S}}\mathbf{j=}\frac{\mathbf{1}}{\mathbf{N}_{\mathbf{j,positive}}}\sum_{\mathbf{i=1}}^{\mathbf{N}} \mathbf{S}_{\mathbf{ij}}\boldsymbol{\cdot}\mathbf{Ⅱ}\mathbf{(}\mathbf{S}_{\mathbf{ij}}\mathbf{>0)}$$

Where:

S*_ij_* represents the severity score for j reported by participant i, measured on a 4-point Likert scale (0 = none, 1 = mild, 2 = moderate, 3 = severe).

Ⅱ(S*_ij_* > 0) is an indicator function that takes the value of 1 if the symptom was present (S*_ij_* > 0) and 0 otherwise.

N*_j, positive_* refers to the number of positive cases for symptom *j*

$\sum_{\boldsymbol{i}=\mathbf{1}}^{\boldsymbol{N}} Ⅱ(\boldsymbol{Sij}＞\mathbf{0})$calculated as the sum of the indicator function across all participants.

In instances where a symptom was not reported by any participant (i.e., $\boldsymbol{N}\boldsymbol{j},\boldsymbol{positive}=\mathbf{0}$), the mean severity score $\boldsymbol{S}_{\boldsymbol{j}}$ was consequently defined as 0.

(4) Total Symptom Burden ($TSB_{k}$)

The total symptom burden for the $k$-th individual was computed as the sum of the severity scores across all assessed symptoms:

$$\boldsymbol{TSB}_{\boldsymbol{k}}\boldsymbol{=}\sum_{\boldsymbol{i=1}}^{N_{t}} S_{i,k}$$

Where:

 $S_{i,k}$ = the severity of the $i$-th symptom for the $k$-th individual,

 $t$ = the total number of symptoms assessed.

$\sum_{i=1}^{N_{s}} S_{i,k}$ = the cumulative severity score for individual $k$ across all symptoms.

## Section S2

1. Prevalence of Exposure (EP)

The EP to a specific risk factor within the study population was calculated as:

$$\boldsymbol{EP=}\frac{\boldsymbol{N}_{\boldsymbol{exp}}}{\boldsymbol{N}}\boldsymbol{\times100\%}$$

Where:

$N_{\text{exp}}$ = number of individuals exposed to the factor

1. Effect Size (ES)

The ES for each risk factor was calculated as the difference in the mean Total Symptom Burden (TSB) between the exposed and unexposed groups, divided by the pooled standard deviation. The formula is expressed as:

$$\boldsymbol{ES=}\frac{\boldsymbol{T}_{\boldsymbol{exp}}\boldsymbol{-}\boldsymbol{T}_{unexp}}{\boldsymbol{S}_{\boldsymbol{p}\boldsymbol{ooled}}}$$

Where:

$\boldsymbol{T}_{\text{exp}}$ = mean TSB of the exposed group

$\boldsymbol{T}_{\text{unexp}}$ = mean TSB of the unexposed group

$\boldsymbol{s}_{\boldsymbol{p}\boldsymbol{ool}\boldsymbol{ed}}$ = pooled standard deviation of TSB between the two groups

1. Breadth of Affected Physiological Systems (BAPS)

This metric represents the number of distinct body systems significantly affected by the risk factor, identified through Mann-Whitney U tests (significance level set at p < 0.05).

1. Composite Risk Score (CRS)

To enable cross-factor comparison, the three metrics were first normalized to a 0-1 scale to eliminate unit differences:

$$\boldsymbol{ES}_{\boldsymbol{norm}}\boldsymbol{=}\frac{\boldsymbol{E}\boldsymbol{S-}\boldsymbol{ES}_{\boldsymbol{min}}}{{\boldsymbol{E}\boldsymbol{S}}_{\boldsymbol{m}\boldsymbol{ax}}\boldsymbol{-}{\boldsymbol{E}\boldsymbol{S}}_{\boldsymbol{m}\boldsymbol{in}}}$$

$$\boldsymbol{EP}_{\boldsymbol{norm}}\boldsymbol{=}\frac{\boldsymbol{E}\boldsymbol{P-}\boldsymbol{EP}_{\boldsymbol{min}}}{{\boldsymbol{E}\boldsymbol{P}}_{\boldsymbol{m}\boldsymbol{ax}}\boldsymbol{-}{\boldsymbol{E}\boldsymbol{P}}_{\boldsymbol{m}\boldsymbol{in}}}$$

$$\boldsymbol{BAPS}_{\boldsymbol{norm}}\boldsymbol{=}\frac{\boldsymbol{SIB-}\boldsymbol{SIB}_{\boldsymbol{min}}}{\boldsymbol{SIB}_{\boldsymbol{m}\boldsymbol{ax}}\boldsymbol{-}\boldsymbol{SIB}_{\boldsymbol{m}\boldsymbol{in}}}$$

The Composite Importance Score was then derived as a weighted sum:

$$\mathbf{CRS}\boldsymbol{=(0}\boldsymbol{.5\times}{\boldsymbol{E}\boldsymbol{S}}_{\boldsymbol{n}\boldsymbol{orm}}\boldsymbol{)+(0}\boldsymbol{.3\times}{\boldsymbol{E}\boldsymbol{P}}_{\boldsymbol{n}\boldsymbol{orm}}\boldsymbol{)+(}\boldsymbol{0.2\times}{\boldsymbol{S}\boldsymbol{IB}}_{\boldsymbol{n}\boldsymbol{orm}}\boldsymbol{)}$$

This weighting scheme prioritizes ES (50%), followed by BAPS (30%) and EP (20%), to identify key targets for intervention.

## Section S3

1. Implementation Rate (IR)

The IR for each protective measure was calculated as follows:

$$\boldsymbol{IR=}\frac{\boldsymbol{N}_{\boldsymbol{imp}}}{\boldsymbol{N}}\boldsymbol{\times100\%}$$

Where:

$N_{\text{imp}}$ = number of individuals who adopted the measure

$N$ = total study population

1. Mean Protective Effectiveness (MPE)

The MPE was calculated specifically among the subpopulation that adopted the measure;

$$\boldsymbol{MPE=}\frac{\sum_{\boldsymbol{k=1}}^{\boldsymbol{N}_{\boldsymbol{imp}}} \boldsymbol{E}_{\boldsymbol{k}}}{\boldsymbol{N}_{\boldsymbol{imp}}}$$

Where:

$E_{k}$ = protective effectiveness score reported by the $\boldsymbol{k}$-th user of the measure

$N_{\text{imp}}$ = number of individuals who adopted the measure

$\sum_{k=1}^{N_{\text{c}}} E_{k}$ = sum of protective effectiveness scores across all users

1. Composite Protective Effectiveness (CPE)

The composite protective effectiveness was determined using a weighted evaluation approach that integrated both utilization rate and protective effectiveness:

$$\boldsymbol{CPE}\boldsymbol{=(}\boldsymbol{0}\boldsymbol{.}\boldsymbol{4}\boldsymbol{\times}\boldsymbol{I}\boldsymbol{R}_{\text{norm}}\boldsymbol{)+(}\boldsymbol{0}\boldsymbol{.}\boldsymbol{6}\boldsymbol{\times}\boldsymbol{MP}\boldsymbol{E}_{\text{norm}}\boldsymbol{)}$$

Where:

$IR_{\text{norm}}$ = normalized utilization rate (0-1 scale)

$MPE_{\text{norm}}$ = normalized mean protective effectiveness (0-1 scale)
The weighting coefficients (0.4 for UR and 0.6 for MPE) were determined through expert consultation, emphasizing protective effectiveness while maintaining consideration for implementation breadth.
